# Resilience among organ donation coordinators: a Canadian mixed-methods study

**DOI:** 10.3389/fpubh.2024.1367546

**Published:** 2024-03-15

**Authors:** Amina R. Silva, Andrea Rochon, Laura Hornby, Ken Lotherington, Lee James, Richard Webster, Ewa Sucha, Aimee Sarti, Sonny Dhanani, Vanessa Silva e Silva

**Affiliations:** ^1^Department of Nursing, Brock University, St. Catharines, ON, Canada; ^2^Department of Nursing, St. Lawrence College, Kingston, ON, Canada; ^3^Canadian Blood Services, Ottawa, ON, Canada; ^4^Children’s Hospital of Eastern Ontario Research Institute, Ottawa, ON, Canada; ^5^Department of Critical Care, The Ottawa Hospital, Ottawa, ON, Canada; ^6^Department of Medicine, University of Ottawa, Ottawa, ON, Canada

**Keywords:** coping strategies, organ donation, organ donor coordinators, protective factors, resilience

## Abstract

**Background:**

Organ and Tissue Donation Coordinators (OTDCs) are key to the success of deceased organ donation processes. However, reduced resilience can leave them susceptible to the incidence of work-related issues and decrease the quality of the care provided. Therefore, this study aimed to examine the extent of resilience and influencing aspects among OTDCs in Canada.

**Methods:**

Mixed-method (QUAN-qual) explanatory sequential design. Quantitative data was collected using an online cross-sectional survey approach with demographic data and the validated scales and analyzed using descriptive and inferential statistics. Qualitative data was collected using a descriptive approach with a semi-structured interview guide and analyzed using content analysis.

**Results:**

One hundred twenty participants responded to the survey, and 39 participants were interviewed. Most participants from the survey were female (82%), registered nurses (97%) and on average 42 years old. The quantitative data revealed that OTDCs had a high level of perceived compassion satisfaction (ProQOL-CS = 36.3) but a resilience score (CD-RISC = 28.5) lower than other groups of healthcare professionals. OTDCs with over a year of experience in the role were more likely to have higher levels of resilience. The qualitative data identified that participants saw resilience as crucial for their work-related well-being. Although coping strategies were identified as a key factor that enhance resilience, many OTDCs reported difficulty in developing healthy coping strategies, and that the use of unhealthy mechanisms (e.g., alcohol and smoking) can result in negative physical consequences (e.g., weight gain) and reduced resilience levels.

**Conclusion:**

Participants reported using a series of coping and protective strategies to help build resilience, but also difficulty in developing healthy mechanisms. The lack of healthy coping strategies were seen as contributing to negative work-related issues (e.g., burnout). Our findings are being used to develop tailored interventions to improve resilience and healthy coping strategies among organ donor coordinators in Canada.

## Introduction

Organ donation supports the life-saving medical intervention of transplantation, and most transplants stem from deceased donors (1). Deceased organ donation is a highly specific and complex field, characterized by a meticulously coordinated sequence of events that must occur seamlessly to ensure successful transplantation (2). Key to this process are Organ and Tissue Donation Coordinators (OTDCs), who facilitate communication and manage procedures among donors, recipients, and healthcare professionals (3). However, the ethical landscape surrounding organ donation is multifaceted. Bioethical considerations in organ donation include obtaining informed consent, ensuring equitable allocation, addressing conflicts of interest, defining death criteria, and preventing commercialization and exploitation (4). Regulatory oversight is paramount, governed by legal frameworks, overseen by Organ Donation Organizations (ODOs), and supported by ethics committees. Compliance with international guidelines further ensures ethical conduct and standardization across jurisdictions (3). Together, adherence to ethical principles and regulatory standards safeguards the integrity and fairness of organ donation, promoting the well-being of donors, recipients, and society.

Despite their key role, OTDCs are often challenged by a unique set of stressors as they navigate emotionally intense situations while managing complex ethical, logistical, and bureaucratic factors (5). Additionally, the time-sensitive nature of the deceased organ donation process due to the urgent need to secure viable organs and tissues for transplantation often adds an extra layer of stress to their work (6). The constant exposure to this inherently demanding and challenging environment can make OTDCs susceptible to work-related issues such as burnout and compassion fatigue, which can lead to reductions in their work-related well-being, increase turnover rates and negatively impact the quality of deceased organ donation processes (6).

A recent scoping review (3) investigated the international literature on work-related issues among OTDCs. Findings from that study highlighted the key role of resilience to manage work-related stressors and how positive aspects of the job, including compassion satisfaction, may help foster inner strength among OTDCs worldwide. Resilience involves the process of effectively adjusting to demanding or challenging life experiences, particularly through mental, emotional, and behavioral adaptability, and the ability to respond flexibly to both external and internal pressures (7). Additionally, the use of healthy coping strategies has been highlighted in the literature as crucial to help healthcare professionals to manage work-related stress and build resilience (8–10). Even though the use of such strategies can help manage work-related stressors and support building resilience among healthcare professionals (11), there is a lack of evidence on this topic in the Canadian context for the specific population of OTDCs (3). Therefore, this study aimed to explore the extent of resilience and influencing aspects among Canadian OTDCs.

## Materials and methods

### Design

In this study, we used a mixed-method (QUAN-qual) explanatory sequential design. The quantitative approach was implemented first with a greater focus on addressing our study aims, followed by a qualitative approach to help further explore the results from the quantitative phase (12). This paper is part of a series of studies from the BRiC (Burnout and Resilience in Organ Donor Coordinators) research program (4). This study received ethics clearance from the Research Ethics Board at the Children’s Hospital of Eastern Ontario (20210076) and additional approvals were obtained from specific ODO sites as needed. To guide the reporting of methods, the STROBE (Strengthening the Reporting of Observational Studies in Epidemiology) for cross-sectional studies (13) and COREQ (Consolidated Criteria for Reporting Qualitative Research) were used (14).

### Study setting and recruitment

We invited all 10 Canadian Organ Donation Organizations (ODOs) to participate in our study, which included programs from the provinces of Alberta, British Columbia, Manitoba, New Brunswick, Newfoundland and Labrador, Nova Scotia, Ontario, Quebec, and Saskatchewan. We used a convenience sampling technique to recruit participants. With support from the ODOs, all OTDCs received an e-mail invitation to take part in our study. Participants were eligible if they were healthcare professionals in the role of an OTDC who worked with deceased multiorgan donation in any Canadian province. The total available sample size was 175 OTDCs from across Canada.

### Data collection

#### Quantitative

For the quantitative phase, we used a cross-sectional survey design approach. The survey was composed of a series of validated instruments that were used to evaluate resilience and compassion satisfaction, along with demographic information to characterize our sample (e.g., age, gender, and education). We used the Connor-Davidson Resilience Scale −10 (CD-RISC-10) (15) to measure the levels of resilience and how individuals are equipped to bounce back after stressful and difficult events. The CD-RISC-10 is a reliable scale with high internal consistency (Cronbach alpha = 0.81–0.94) that has been validated among health professionals and is comprised of 10 out of the 25 items from the original scale (16). Additionally, the measure of Compassion Satisfaction (CS) from the Professional Quality of Life Measure (ProQOL) was used to assess the positive effects of helping others who experience suffering. This is a reliable scale with high internal consistency (Cronbach alpha for CS = 0.88) that has been validated among health professionals (17). Assessing compassion satisfaction provides valuable insights into the positive aspects of OTDCS experiences, offering a nuanced perspective on how fulfilling aspects of their work may contribute to effective coping strategies and bolster overall resilience in the face of the inherent challenges within the field of deceased organ donation.

The survey was hosted online and administered by Canadian Blood Services using a third-party licensed product (^®I^nterceptum). After participants received the e-mail invite, they accessed the survey and consented to participate prior to having access to the questions. Participants had a total of 60 days to complete the survey and reminders were sent every 15 days (18). All participants who completed the survey received a $50 gift card.

#### Qualitative

We used a descriptive design for the qualitative data as this approach can be used to explore issues that are present in healthcare settings (19, 20). Participants were asked in the survey if they would have an interest in participating in the qualitative portion and we contacted those who agreed for the interviews. We used a semi-structured interview guide (Supplementary Table S1) that was developed, and pilot tested with 5 # OTDCs and as there were no considerable changes made to the guide, included the pilot test participants in the study sample. All interviews were conducted by a female member of the research team (A.R.S., RN, and Ph.D.), who had experience in qualitative research, online using the video conference feature hosted at Zoom^®^, and individual interviews ranged from 29 to 146 min. The interviews were performed until data saturation by repetition of findings was identified in participants’ responses from each province. All interviews were video/audio recorded. The reflexive coding process in this study involved the researcher (A.R.S., RN, and Ph.D.) using descriptive and reflexive field notes during interviews to document their own thoughts and perceptions. After transcription, data analysis proceeded iteratively with open coding techniques, constantly reflecting on the researcher’s biases and assumptions. Codes were organized into themes and subthemes, with constant comparison techniques ensuring rigor. Reflexive memos documented shifts in understanding throughout the process, enhancing the credibility of the findings by acknowledging the researcher’s subjective influence on interpretations.

### Data analysis

#### Quantitative

Statistical analyses were performed using ^®^R version 4.0.5 (21). Descriptive statistics (e.g., mean and frequency) were used to characterize the sample demographics and the tool scores. We used inferential statistics (e.g., regression analysis) to establish relationships among the variables sex, years in the role, age, and weekly hours worked. Those variables were defined based on previous literature and the relevance to address the study research questions (3). The statistical analysis was performed by professional statisticians with experience in health research (R.W. and E.S.).

#### Qualitative

After interviews, the recordings were transcribed verbatim by a professional transcriptionist; records were not returned to participants. For participants who opted to be interviewed in French, a translation was performed in the transcription of the recording by a professional translator. The data was imported into a qualitative data analysis software ^®^NVivo (QRS international, AU) to organize and manage the data. A content data analysis approach was performed by one member of the team (A.R.S.) and peer reviewed (A.R. and V.S.S.) to achieve consensus across the data and quantify the reports as needed. The processes of interview, transcription, and data analysis occurred concurrently as this enabled assessment of the need to revise the interview guide and/or continue interviews for data saturation (22). Reflexive field notes were used to support the coding of interviews but those were not coded separately. Lastly, triangulation involved a sequential approach where quantitative survey results informed the development of qualitative semi-structured interview guide to gain insights from in-depth interviews. The integration of both qualitative and quantitative findings during the interpretation phase allowed for a robust understanding of the complex interplay among the concepts of interest in this study.

## Results

A total of 120 OTDCs (68.6% of the available Canadian sample) responded to the survey, and 39 (22.3%) of respondents were interviewed. Characteristics of the survey sample can be found in Table 1 (participants from interviews were not linked to the quantitative data to protect their confidentiality). Most OTDCs were female (*n* = 98; 82%), registered nurses (*n* = 117; 97%) and were on average 41.7 ± 10.2 (25–67) years old and have worked in the role for over a year (*n* = 84%).

**Table 1:** Respondents characteristics of survey.

Characteristic	*N*(%)
Sex	
Female	98 (82.4%)
Male	21 (17.6%)
*Missing*	1 (0.8%)
Age	
Mean ± *SD* (min–max)	41.7 ± 10.2 (25–67)
Education (combined)	
Registered nurse	117 (97.5%)
Respiratory therapist	3 (2.5%)
Years working as OTDC	
1–6 months	3 (2.5%)
6 months–1 year	16 (13.6%)
More than 1 year	99 (83.9%)
*Missing*	2 (1.7%)

### Quantitative

#### Resilience – CD-RISC scale

When measuring resilience with the CD-RISC scale, our sample had a mean score of 28.5 ± 5.62, which indicates a moderate to low level of resilience (Table 2). We also assessed the data to evaluate the relationship between the mean of the CD-RISC scale and the covariates defined as: sex (*p* = 0.807); years in the role (*p* = 0.006); age (*r* = 0.15); and weekly hours worked (*r* = 0.08) using a regression analysis (Table 3). Years in the OTDC’ role was the only covariate that presented a statistically significant relationship (*p* = 0.006) where professionals with over a year of experience in the role were more likely to have higher levels of resilience.

**Table 2:** ProQOL-CS and CD-RISC scores.

	ProQOL.CS.score	CD.RISC.score
Mean ± *SD*	36.3 ± 6.26	28.5 ± 5.62
Median [min, max]	37.0 [16.0, 49.0]	29.0 [10.0, 40.0]
Missing	7 (5.8%)	2 (1.7%)

**Table 3:** Regression analysis using the CD-RISC score.

CD-RISC score^*^	Variable			
Sex	Female (*N* = 98)		Male (*N* = 21)	*P*-value
Mean (*SD*)	28.4 (5.87)		29.0 (4.26)	0.807
Median [min, max]	29.0 [10.0, 40.0]		29.0 [23.0, 39.0]	
Missing	2 (2.0%)		0 (0%)	
Years working as an OTDC	1 year or less (*N* = 19)		More than 1 year (*N* = 99)	*P*-value
Mean (*SD*)	24.9 (5.47)		29.2 (5.47)	0.006
Median [min, max]	27.0 [10.0, 31.0]		30.0 [17.0, 40.0]	
Missing	1 (5.3%)		1 (1.0%)	
Difficulty of last case managed	Extremely difficult (*N* = 54)	Fairly difficult (*N* = 28)	Not difficult at all (*N* = 37)	*P*-value
Mean (*SD*)	28.1 (5.63)	28.4 (5.56)	29.0 (5.51)	0.819
Median [min, max]	29.0 [10.0, 37.0]	28.0 [14.0, 40.0]	28.0 [19.0, 40.0]	
Missing	1 (1.9%)	1 (3.6%)	0 (0%)	

^*^OR = 0.91; CI 95% = 0.87, 1.07; *p* = 0.5.

#### Compassion satisfaction—ProQOL-CS

When assessing for compassion satisfaction, according to the ProQOL-CS scale, the mean score was 36.3 ± 6.26. For this scale, scores below 23 can indicate a negative perception (e.g., low compassion satisfaction) regarding that domain. OTDCs in our study had a high level of perceived compassion satisfaction (Table 2).

#### Qualitative

In the interview data, we have identified two major categories that described the meaning and role of resilience for OTDCs and strategies used to help manage work-related stressors: (1) Resilience: Building Inner Strength; and (2) Coping and Protective Strategies: Navigating Adversity.

#### Resilience: building inner strength

Resilience was perceived by participants as a fundamental aspect to help maintain their work-related well-being. Participants defined resilience as “*the ability to bounce back*” (P10), “*keep going when things do not always go right*” (P3), “*different ways you are able to cope*” (P4), “*being able to ask for help*” (P4), “*deal with stress and hardship*” (P9) and “*come back and do it over and over and over again*” (P29). All participants, when questioned, reported seeing themselves as a resilient person and that the OTDC role “*helped [them] to become more resilient*” (P3) as they are constantly “*pushed out of comfort zone*” (P3) and so they get used to the challenges. As one participant reported: “*Every time my pager goes off someone has died. That does not bother me any more now […] it’s become a job in many respects and, and you’ll get the ones that crack through, but I think that […] having that resilience or having a little thicker skin to some tragic circumstances*” (P28). Still, some participants reported that “*pushing through things is not, to some detriment, is not resiliency*” (P2).

Participants believed that there is a series of personal attributes that can make a person more resilient, such as personality, support system, and previous professional experience. Past exposure to trauma was also reported as a source of resilience, as mentioned by one participant: “*I know people who have gone through like death in their own families, really bad divorces,* var*ious kinds of emotional abuse, but that’s made them resilient (…) they can just keep going*” (P3). Still, participants believed that “*without having a foundation of support and structure it is hard to feel resilient, it is hard to feel strong*” (P4). Lastly, OTDCs also reported that effective coping and protective strategies, as well as a sense of job satisfaction, are essential in helping build resilience.

#### Coping and protective strategies: navigating adversity

The use of coping and protective strategies was discussed interchangeably in our sample, and OTDCs highlighted those factors as pivotal in managing stressors, enhancing their mental health, and building resilience. The most reported method of coping among participants was the use of informal debriefing or venting. OTDCs stated that colleagues are the best source of informal support because “*they are the only ones who really understand*” (P3) what they go through. Oftentimes, OTDCs also reach out to colleagues to seek information on how to proceed in certain situations or even to ask for support when they feel overwhelmed, as reported by one participant: “*I did call my other colleagues, just to talk about it and just say, I do not think that I can be here and do this case anymore because it’s really, really affecting me”* (P1). However, participants also expressed concerns that informal debriefing can leave colleagues exposed to secondary traumatic stress “*like, we [OTDCs] are like slamming at each other, like, it wasn’t always good*,” as well as the worry of being “*too vulnerable with co-workers*” (P4) while debriefing emotionally charged situations. The complete list of healthy coping and protective strategies mentioned by participants can be found in Table 4 along with a sample quote and the number of participants that reported each strategy.

**Table 4:** Coping and protective healthy strategies reported with sample quotes.

Strategy	Participants	Sample quote
Debriefing or venting	*N* = 37	“*I want to say as a group of coordinators we do have a good relationship with each other, we know when someone is not doing well, we have started noticing certain things and then certain OTDC’s will be the one that says ‘listen, I’ll be talking to them later.’ So, there’s always someone for someone else, that’s what I – so, I do find that we can see when our colleagues aren’t doing well.”* (P4)
Family or friends support	*N* = 19	*“I speak with my partner a little bit but he’s not in healthcare, so he does not completely understand which is perfect because then we do not talk about healthcare all the time.”* (P16)
Taking a break	*N* = 18	*“So you know, sometimes I’ll just take… give myself a moment, excuse myself or just explain to them quickly that, you know, this is emotional for me as well and, you know, just give everybody a moment to reorganize my thoughts and then take a deep breath and just try to keep going.”* (P17)
Boundaries	*N* = 16	*“It’s really important to have very firm boundaries and say I’m not on call today. I have plans with my family. I’m not available to help even though it’s busy. And like even establishing and maintaining those boundaries is its own source of stress because you know (…) you are saying no, that means that somebody else is under stress because of it.”* (P23)
Physical activities	*N* = 13	*“I started running and I found I was just so empowered after. There was something that it did to my body, like it’s like hemostasis afterwards. So sometimes I’ll go for a run if I have a really distressing day and put my headphones in, listen to some music and I feel a lot better after that as well.”* (P5)
Detachment	*N* = 8	*“I probably detach from most where I try to just keep, you know, obviously still be as compassionate as I can with the families but learning limited information. As limited as I need to get by, because you know, that’s hard to carry their grief, right? Like it’s challenging.”* (P21)
Self-reflection	*N* = 6	*“I usually try to get up and get away and like distract myself and kind of also try to rationale it, like I understand that he [donor family member] was upset, and I could understand, you know, just going through that in my head. Which kind of helps somewhat.”* (P11)
Going outdoors	*N* = 3	*“Being outdoors is kind of when I can just kind of clear my head or go throw the ball around with my kids or whatever.”* (P2)
Media use	*N* = 3	*“I’ll just go to bed really early, you know, watch some [TV shows], and have some popcorn and catch up on sleep.”* (P5)
Focus on positive side	*N* = 2	*“When I see something bad, I become more grateful for what I have.”* (P6) *“when I’m feeling stress, probably just in general, I make sure that I do spend some time with recipients here on the island. (…) So, I just try and make sure that I remember what the big picture is and that the little parts might be difficult, but the big picture is the big picture.”* (P16)
Alternative medicine	*N* = 1	*“I do believe in my essential oils and I love them to death (…) I manage to have some sort of candles everywhere that I like that he says okay to but if I have an oil diffuser that’s only in my office”* (P37)

Participants also reported that some aspects of their job can help improve their work-related well-being. For instance, some OTDCs mentioned that they are at “*the top of the pay scale*” (P3) and that no other bedside nursing job would have similar monetary compensation, which motivates them in staying in the role. The possibility of working from home during working days was also seen as a positive aspect since they can be “*more productive to be at home and to not have to commute*” (P12), as well as it increases their sense of freedom and improve mental health.

Employee Assistance Programs (EAPs), which provides mental health support along with other benefits to employees, and organizational counselors were also frequently mentioned by participants, but with ambivalent feelings. When effective, counselors can help OTDCs in managing work-related stressors, as mentioned by a participant: “*I talk to they [counselor] at least a couple times a month just to check in, just to make sure everything is okay (…) they are a really great resource for us*” (P5). However, some participants reported negative experiences where during debriefing sections with counselors: “*people got punished for sharing their thoughts, their emotions or whatever and as a result people were not trusting anymore*” (P4) referring to the use of debriefing information to evaluate performance by leadership. Additionally, many participants reported difficulties in accessing EAP resources as they had “*no idea how to access the EAP*” (P1) and felt uncomfortable asking for such information as people would start questioning them on “*what’s wrong, why do you need to talk to this person?*” (P1). Participants also felt it was not worth using the EAP due to the limit of sessions that the benefit would cover where “*once you go four times, it’s kind of like okay, do I keep paying for this or do I wait until the next year until I can start going again*” (P13). While others did not see the support from EAP as valuable as the counselor did not understand their area of work as one participant stated “*I had to repeat myself so many times that I got so tired of repeating myself. So, I just called in sick all the time”* (P4). Still, some participants had negative experiences with the counselors that instead of providing support for self-development were suggesting they leave the job: *“I’ve seen a couple of counselors, you know, over the years and every single one of them would say you need to leave your job. And I would think well that’s not very effective counseling. (…) But I wasn’t willing to have to leave a job”* (P14).

The use of unhealthy strategies to help cope with job stressors and negative consequences were also mentioned by some participants where they: “*smoke a little bit here and there*” (P16), “*eat fast food*” (P21), “*drinking some*” (P14, P26, and P32) and consequently “*put on weight*” (P16) since starting the role. Furthermore, despite participants understanding of the importance of applying effective coping and protective strategies, many participants reported challenges in developing those, as one participant expressed: “*I’m not the best coper in the world and I will push through a lot of things until I break*” (P2). The lack of coping strategies was also seen by participants as a potential cause of work-related issues among OTDCs: “*I think that [lack of coping strategies] is probably one of the reasons why there’s so much burnout with coordinators, (…) we are so tired after a case and then what do we do to look after ourselves?*” (P21).

## Discussion

In this mixed-methods study, we sought to examine the extent of resilience and influencing aspects among OTDCs in Canada. OTDCs believe that resilience is a reflection of a multifactorial process that includes personal attributes, past professional experience, previous exposure to trauma, and ability to cope. Participants also reported using a series of coping and protective strategies to help build resilience, with *debriefing and/or venting*, *family and/or friends support*, and *taking a break* being the most often reported. Our results lend insight into OTDCs’ experiences and provide valuable information on how to best support OTDCs to enhance their work-related well-being.

Although all OTDCs reported feeling resilient during the qualitative interviews, the quantitative analysis revealed that our sample exhibited a lower level of resilience when compared to previous normative samples of both healthcare professionals and the general population (23). This disparity may be attributed to the multifaceted nature of resilience and contextual influences that may have led OTDCs to perceive themselves as more resilient than the actual resilience level measured in the resilience scale results. Additionally, our study revealed that while OTDCs faced high levels of work-related stressors, they exhibited an increased level of compassion satisfaction—a clear indication of the satisfaction derived from performing their work effectively and the positive outcomes from the donation process (e.g., supporting families, transplant recipients). This finding aligns with previous studies (3), which also reported high job satisfaction among OTDCs, despite encountering significant work-related challenges.

The use of effective coping and protective strategies were also reported by participants as key to foster inner strength. Similarly, there are substantial meta-analytical findings (8–10) supporting the use of those strategies to help healthcare professionals to manage work-related stressors and foster resilience. Still, despite the importance of such management strategies, our results, which are echoed by the existing literature among Canadian OTDCs (3, 5, 6), emphasized that OTDCs often report challenges in developing healthy coping and protective strategies and that they usually keep pushing through situations without taking proper time to reflect and cope due to the highly complex and demanding environments they work in. Even though our study was focused specifically on Canadian OTDCs, the international literature similarly reported on the importance of management strategies to enhance work-related wellbeing and resilience (3, 24).

Among the most cited source of coping for OTDCs in this study is the use of informal debriefing and venting. The use of debriefing has been suggested as an important resource for coping among healthcare professionals (5, 25, 26). However, our sample expressed concerns that informal debriefing may leave individuals susceptible to secondary traumatic stress from listening to others’ emotionally charged cases. Despite the importance of such findings, to the best of our knowledge, the existing literature does not provide guidance on addressing this matter, and further research should focus on that aspect. Lastly, a substantial number of participants (*n* = 8) who reported detachment as a coping strategy; however, it is important to emphasize that existing evidence suggests detachment as a symptom of compassion fatigue (27). This finding reinforces the notion that OTDCs might be experiencing work-related issues such as burnout or compassion fatigue without fully recognizing the signs and symptoms, which has also been identified in past studies (3).

Existing literature demonstrated that tailored training in deceased organ donation on how to develop coping strategies to build inner resilience can positively impact the sense of preparedness of coordinators, and patient outcomes (28–30). Despite evidence on the needs and potential impact of tailored training for coordinators, there is still a lack of initiatives in the Canadian scenario (30). Recognizing this need for Canadian OTDCs, our research team, along with national agencies (Canadian Blood Services), is working on an interventional studies to support OTDCs in developing healthy coping strategies and enhance their resilience levels; which can ultimately improve deceased organ donation outcomes.

### Limitations

While our findings hold promise for informing tailored interventions aimed at enhancing the deceased organ donation process, it is essential to recognize certain limitations. The absence of measures pertaining to coping strategies in our cross-sectional design aligns with our initial objectives, yet their inclusion could bolster the robustness of our conclusions. Moreover, our sample primarily comprises Canadian OTDCs, thereby restricting the generalizability of our findings to other geographical regions. Additionally, while the provision of a framework to support OTDCs in bolstering their resilience would be valuable for readers, such insights are part of an ongoing project within our research team, and integration of these results is currently unavailable. Lastly, while most participants were active in the role of OTDCs during the study implementation, learning the perspectives of former OTDCs on those topics could help to understand the reasons that may have led them to leave their role.

## Conclusion

This study lend insight into OTDCs’ perceptions of resilience and the contribution of external aspects (e.g., past experience) and personal coping strategies (e.g., social support) to enhance their resilience levels. Our findings provide valuable information to organizations and researchers on how to effectively support OTDCs in cultivating resilience. For instance, through the supporting the development of healthy coping strategies to enhance OTDCs’ resilience. By improving OTDCs resilience and work-related wellbeing, those interventions may also enhance the overall quality of deceased organ donation processes. While our study focused on Canada, the work-related stressors faced by OTDCs worldwide are similar as evidenced by international literature, making our results transferable to international contexts. Lastly, interventional studies are currently needed to help address the issues identified in our study, including how to develop healthy coping and protective strategies to enhance resilience levels and mitigate the effects of work-related stressors, as well as investigations on secondary traumatic stress due to the exposure during informal debriefing with colleagues.

## Data availability statement

The original contributions presented in the study are included in the article/Supplementary material, further inquiries can be directed to the corresponding author.

## Ethics statement

The studies involving humans were approved by Children's Hospital of Eastern Ontario Research Institute. The studies were conducted in accordance with the local legislation and institutional requirements. The participants provided their written informed consent to participate in this study.

## Author contributions

ARS: Conceptualization, Data curation, Formal analysis, Funding acquisition, Investigation, Methodology, Project administration, Resources, Software, Supervision, Validation, Visualization, Writing – original draft, Writing – review & editing. AR: Conceptualization, Data curation, Formal analysis, Investigation, Methodology, Visualization, Writing – review & editing. LH: Conceptualization, Investigation, Methodology, Project administration, Resources, Supervision, Writing – review & editing. KL: Conceptualization, Funding acquisition, Investigation, Project administration, Resources, Supervision, Validation, Visualization, Writing – review & editing. LJ: Investigation, Project administration, Resources, Supervision, Writing – review & editing. RW: Formal analysis, Software, Validation, Writing – review & editing. ES: Formal analysis, Software, Validation, Writing – review & editing. AS: Data curation, Formal analysis, Writing – review & editing. SD: Visualization, Writing – review & editing, Conceptualization, Data curation, Funding acquisition, Investigation, Methodology, Project administration, Resources, Supervision, Validation. VS: Conceptualization, Data curation, Formal analysis, Funding acquisition, Investigation, Methodology, Project administration, Resources, Software, Supervision, Validation, Visualization, Writing – original draft, Writing – review & editing.
