# Supplementary material for: Resilience among organ donation coordinators: a Canadian mixed-methods study
Source: Front Public Health. 2024 Mar 15;12:1367546. doi: 10.3389/fpubh.2024.1367546 (PMC10978649; doi:10.3389/fpubh.2024.1367546)
Supplement: Supplementary file 1 [file Table_1.DOCX]

**Table S1 – Example of interview questions**

| **Semi-structured interview guide** |
| --- |
| What does resilience mean to you? |
| What do you believe are predictors of resilience? |
| What coping strategies do you employ to cope with the demands of your job? |
| How do you describe your control over emotions during donation events? |
